# Time-saving method for directly amplifying and capturing a minimal amount of pancreatic tumor-derived mutations from fine-needle aspirates using digital PCR

**DOI:** 10.1038/s41598-020-69221-6

**Published:** 2020-07-23

**Authors:** Yusuke Ono, Akihiro Hayashi, Chiho Maeda, Mayumi Suzuki, Reona Wada, Hiroki Sato, Hidemasa Kawabata, Tetsuhiro Okada, Takuma Goto, Hidenori Karasaki, Yusuke Mizukami, Toshikatsu Okumura

**Affiliations:** 1 Institute of Biomedical Research, Sapporo Higashi Tokushukai Hospital, Sapporo, Hokkaido 065-0033 Japan; 2 Division of Gastroenterology and Hepatology/Oncology, Department of Medicine, Asahikawa Medical University, Asahikawa, Hokkaido 078-8510 Japan

**Keywords:** Diagnostic markers, Gastroenterology

## Abstract

It is challenging to secure a cytopathologic diagnosis using minute amounts of tumor fluids and tissue fragments. Hence, we developed a rapid, accurate, low-cost method for detecting tumor cell-derived DNA from limited amounts of specimens and samples with a low tumor cellularity, to detect *KRAS* mutations in pancreatic ductal carcinomas (PDA) using digital PCR (dPCR). The core invention is based on the suspension of tumor samples in pure water, which causes an osmotic burst; the crude suspension could be directly subjected to emulsion PCR in the platform. We examined the feasibility of this process using needle aspirates from surgically resected pancreatic tumor specimens (n = 12). We successfully amplified and detected mutant *KRAS* in 11 of 12 tumor samples harboring the mutation; the positive mutation frequency was as low as 0.8%. We used residual specimens from fine-needle aspiration/biopsy and needle flush processes (n = 10) for method validation. In 9 of 10 oncogenic *KRAS* pancreatic tumor samples, the "water-burst" method resulted in a positive mutation call. We describe a dPCR-based, super-sensitive screening protocol for determining *KRAS* mutation availability using tiny needle aspirates from PDAs processed using simple steps. This method might enable pathologists to secure a more accurate, minimally invasive diagnosis using minute tissue fragments.

## Introduction

It is challenging to acquire histological evidence regarding solid tumors non-invasively; this hamper clinical management decisions that need to be made at appropriate time points. Fine-needle aspiration (FNA) is a standard procedure for collecting tumor tissues; however, there are cases where inadequate sampling resulted in false negative results^1^. This technical issue might be highlighted when tumors, including pancreatic ductal adenocarcinomas (PDAs), which have a low tumor cell content, are targeted. Because of the invasiveness of needle-assisted cytology and biopsy as well as the potential for tumor cell dissemination, albeit at a low incidence, the frequent repetition of the procedure is not generally recommended^2,3^.

The assessment of the tumor grade and histological type is an essential task for pathologists; however, there are possibilities of inter-pathologist diagnostic disagreement^4^. Information regarding the expression levels of specific tumorigenesis-associated proteins in routine clinical practice enables pathologists to reach a consensus on the matter^5^. Limited amounts of specimens can also be an obstacle for performing additional molecular analysis, which emphasizes the necessity of alternative tools that provide evidence regarding malignant tumors^6^.

A robust solution might involve the detection of frequently mutated genes in a specific type of cancer^7^. For instance, in human PDAs, the *KRAS* gene is ubiquitously mutated, and in over 90–95% of patients, lesions emerged because of oncogenic events at the earliest periods of the tumorigenesis process^8^. Another initiating driver mutation in *KRAS* has been reported in colorectal (40%) and lung adenocarcinomas (15–20%). Mutations in other types of tumors, including *BRAF* mutations in melanomas (50–90%) and papillary thyroid carcinomas (50%), *EGFR* mutations in lung cancer, and *PIK3CA* mutations in colorectal and breast cancer might be used as genetic markers for the early identification of malignant tumors^9–12^.

Recent technological advances in genetics such as sequencing and PCR-based genetic analysis might allow the super-sensitive and absolute quantification of very low levels of mutant alleles, even in a small yield of tumor samples with shallow cellular content^13^. Here, we sought to further develop a new digital PCR (dPCR) protocol using tissues collected from pancreatic tumors by FNA, which allows for the detection of genetic mutations in small amounts of specimens. In pre-clinical settings, by obtaining tumor specimens right after resection, a high accuracy of detection of tumor cell-derived DNA via dPCR was achieved. By eliminating the genomic DNA purification process, the sample could be processed in a simple and rapid manner and subsequent analysis could be conducted; this may support the routine clinical diagnosis.

## Results

### Development of a method to detect the minimal copy number of tumor-derived mutant *KRAS*

**Figure 1:**
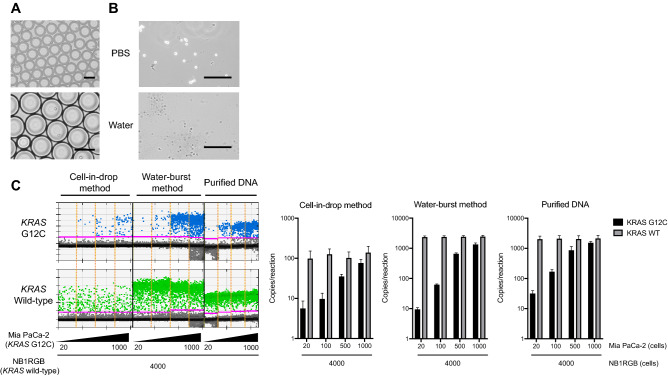
Experimental results using cell lines to improve the dPCR method for the highly sensitive mutation analysis of simple prepared samples. (**A**) Encapsulated cells in dPCR droplets. Cells were collected, resuspended in dPCR reaction solution, and mixed with droplet generation oil using the QX200 droplet generator. Scale bars; 200 μm. (**B**) Cells were burst using pure water. Cells were collected and resuspended in nuclease-free water, which caused an osmotic burst of cells, and genomic DNA was released into the water. The “crude” solution, including gDNA, was used as the dPCR template. Scale bars; 500 μm. (**C**) The DPCR assay was performed using the two novel DNA preparation methods, without a purification step. *KRAS* wild-type (Fibroblast; NB1RGB) and *KRAS* G12C (PDA; MIA PaCa-2) cells were mixed with several dilution series (*left panel*). The wild-type or G12C mutation in *KRAS* was detected using the QX200 droplet reader, as compared to the conventional DNA preparation method using commercial purification kits (see details in “Methods”). The *KRAS* copy number of wild-type or G12C measured by QuantaSoft software (*right panel*).

We investigated a method for detecting mutations in tiny tissue samples using absolute quantification via dPCR. To prepare input DNA from the samples, we first tested two methods, to avoid losses during the nuclear purification step. In the first method, cells/tissues were encapsulated using the droplet generator, and the PCR reaction was then directly performed. We performed serial dilution using two different cell lines, i.e. MIA PaCa-2 (homozygous *KRAS* G12C) and NB1RGB (wild-type *KRAS*). The ratios of mutants to wild-type genes in cell mixtures were 20:4,000, 100:4,000, 500:4,000, and 1,000:4,000. The cells suspended in 4 µL of PBS were directly enclosed within emulsion drops (Fig. 1A), using the QX200 system, and then used for the dPCR mutation detection assay. As shown in Fig. 1C, the frequency of detection of mutations after the capture of the enclosed cells was modest (12.9% in *KRAS* mutant cells or 2.9% in wild-type *KRAS* cells, on average).

We, therefore, examined the alternative method, where cells were collected and resuspended in nuclease-free water, which caused an osmotic burst of collected cells; this could cause genomic DNA to be released into the liquid fraction (Fig. 1B). The “crude” DNA was then directly utilized as the dPCR template without performing the DNA purification step (around 30 min); hence, throughout the assay, we could determine the *KRAS* mutation status in fresh tumor samples within 2.5 h (a few minutes of preparation of tumor-derived DNA before processing dPCR). The dPCR reaction proceeded successfully even with impure DNA, and the detection of the *KRAS* copy number was comparable to that for the sample prepared through conventional DNA purification (Fig. 1C). These results suggested that the “water-burst” method could be used to perform both timesaving preparation steps and achieve high-efficiency detection of small numbers of DNA copies.

### Mutation detection analysis using fresh needle-aspirated tissues from resected pancreatic neoplasia

Next, we examined the feasibility of detecting *KRAS* mutants using a tiny amount of resected tumor tissue. Twelve patients with pancreatic tumors were enrolled, and the needle aspirates from the tumor and the non-tumor areas of the resected specimens were analyzed. The aspirates were suspended in nucleus-free water following storage and spin-down. In the dPCR assay, *KRAS* G12/G13 mutations were found in the PDA from 10 patients, including IPMN-associated pancreatic cancers; 1 PDA patient exhibited mutant *KRAS* Q61H. In contrast, no *KRAS* mutation was detected in tumors in a patient with a pancreatic neuroendocrine tumor (Table 1). Multiple *KRAS* mutations were found in the tumor obtained from one patient with IPMN-associated carcinoma.

**Table 1:** *KRAS* mutation analysis in punctured specimens obtained from resected pancreatic tumor tissues.

Patient	Age	Sex	Surgically resected specimens				Fresh tissue aspirates			
			Pathological diagnosis	Histological type	Tumor cellularity*	Mutation profiles of *KRAS* (%MAF on targeted sequensing)	Wild-type *KRAS* (copy/reaction)	G12/G13 mutant *KRAS* (copy/reaction)	*KRAS* G12/G13 MAF (%)	Results of *KRAS* G12/G13 screening in the dPCR assay**
1	68	F	PDA	Mod	Mod	G12V (12.8)	2,130	18	0.82	Positive
2	76	M	Acinar cell carcinoma	N/A	High	G12D (16.6)	640	112	15.97	Positive
3	69	F	IPMN-associated PDA	Mod	Mod	G12D (18.8)	538	68	10.84	Positive
4	77	M	IPMN-associated PDA	Well	Low	G12D (6.6)	5,100	100	1.92	Positive
5	54	F	PDA	Por	Low	Q61H (31.5)	11,980	10	0.09	Negative (Q61mutation 13.6%***)
6	76	M	IPMN	Low-grade	Mod	G12D (24.7), G12V (21.4), G12S (1.0)	400	22	5.21	Positive
7	81	F	PDA	Mod	Mod	G12V (5.0)	105,580	9,383	8.16	Positive
8	69	M	PDA	Por	Mod	G12D (43.8)	15,500	1,220	7.30	Positive
9	65	M	IPMN	High-grade	Mod	G12V (39.9)	5,053	2,413	32.32	Positive
10	61	F	P-NET	G-1	High	WT	2,025	4	0.20	Negative
11	71	M	IPMN-associated PDA	Por	Mod	G12D (7.4)	17,560	14,910	45.92	Positive
12	70	M	IPMN	High-grade	Mod	G12V (18.6)	90	78	46.43	Positive

*IPMN* intraductal papillary mucinous neoplasm, *MAF* mutant allele frequency, *PDA* pancreatic ductal adenocarcinoma, *P-NET* pancreatic neuroendocrine tumor.

*Tumor cellularity; low, < 10%, medium, 10–30%, high, > 30%.

**Mutation detection assay was performed using ddPCR KRAS G12/G13 Screening Multiplex Kit (Bio-Rad); cut-off > 0.2%.

***For the case with KRAS Q61 positive lesion determined by target sequencing, additional dPCR was performed by ddPCR KRAS Q61 Screening Kit (Bio-Rad); cut-off > 0.5%.

Using the “water-burst” method, we successfully detected the *KRAS* G12/G13 mutations in the fresh needle aspirates from 10 PDAs with corresponding mutations. Besides, the *KRAS* Q61 mutation was found in 1 sample from a patient exhibiting *KRAS* Q61H mutation, while the number of *KRAS* G12/G13 mutations was below the cut-off value (Table 1, Supplementary Figure). We found a 100% concordance in *KRAS* mutations in a small tumor cohort including a sample with wild-type *KRAS*. The lowest frequency in the mutation to wild-type in these patients was 0.82% (mutation allele frequency in the primary tumor lesion was 12.8%; Table1).

### Detection of *KRAS* mutations via dPCR using residual tissues of endoscopic biopsy samples

**Figure 2:**
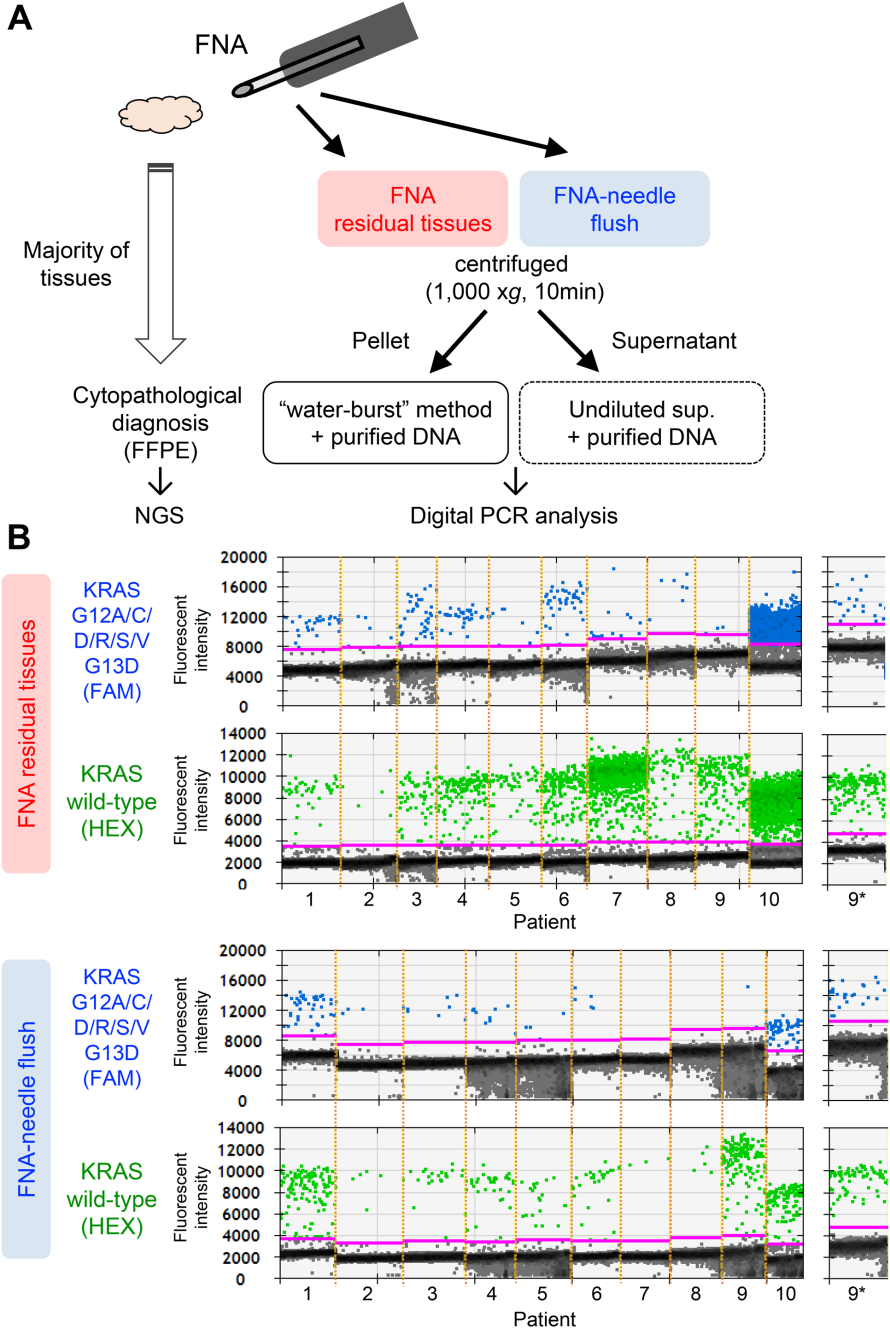
*KRAS* mutation analysis using FNA residual tissues via the “water-burst” sample preparation method. (**A**) Workflow for sample collection and DNA preparation. After submitting patient specimens obtained via FNA for cytopathological diagnosis, residual specimens and the needle washing solution were collected, centrifuged, and separated into a pellet and supernatant. DNA was prepared by the “water-burst” method, in which the precipitate was centrifuged and suspended in water, and dPCR analysis was then performed. The supernatant was directly subjected to the dPCR reaction. These methods do not require DNA purification, and it takes about 2.5 h to obtain genetic information after the collection of a sample. Purified DNA was also subjected to the assay as control (see Table 2). (**B**) DPCR plot of the *KRAS* G12/G13 mutation assay in the collected tissues were resuspended using water (*left large panels*). The plot graph shows the pattern of detection of *KRAS* tissues obtained via centrifugation from FNA residual tissues or needle rinsed fluids. The threshold (solid pink line) was manually set to extend to an amplitude of 2,000 or 1,000 (FAM mutant or HEX wild-type probe) above the maximum background intensity value. The asterisk indicates the results for the *KRAS* Q61 mutation assay for patient 9 (*right small panels*; see Table 2).

We attempted to validate the capture of dPCR-based driver mutations via the “water-burst” method using a residual piece of tumor tissue in the FNA needle. After submitting pancreatic tumor biopsy specimens to the pathology laboratory, minimal amounts of the remaining samples were collected to test the “water-burst” method, by scratching the residual tissues from Petri dishes and flushing the needle with the stabilizing solution (Fig. 2A). The fluid was preserved, shipped, and centrifuged before genetic analysis. Then, the pellets suspended in water and the supernatants were analyzed via the dPCR assay, which targeted *KRAS* mutations. In 9 of 10 patients, we found *KRAS* mutations in residual tissues (G12/G13 mutants in 8 patients, Q61 mutant in 1 patient) obtained after FNA. In 7 specimens of needle-rinsed fluids, we detected G12/G13 mutations in 6 patients, and Q61 mutation in 1 patient (Table 2, Fig. 2B). In patient 7, who was diagnosed with a pancreatic acinar cell carcinoma exhibiting no *KRAS* mutations, the level of the *KRAS* G12/G13 variant was found to be approximately similar to the detection limit of the screening kit (0.2%). In contrast, the mutation allele frequency in other samples with mutant *KRAS* was over 10%. Either the residual tissue or needle flush part of the FNA samples was also analyzed using dPCR assay following DNA purification (Table 2). A strong correlation was observed between amplified *KRAS* copy numbers and the amount of template DNA (Supplementary Table 2 and Supplementary Fig. 2). The “water-burst” assay using pellets from FNA residual tissue showed a *KRAS* mutant allele frequency equivalent to that of purified DNA except for patient 2, whereas the supernatants required DNA purification.

**Table 2:** Mutation analysis using residual tissues in FNA needles.

Patient	Age	Sex	FNA (Tumor specimen)			FNA residual tissue				FNA needle flush		
						Pellet		Supernatant		Pellet	Supernatant	
			Pathological diagnosis	Tumor cellularity*	Tumor genotypes (targeted sequensing)	Water-burst (%MAF)**	Purified DNA (%MAF)**	Unpurified (%MAF)***	Purified DNA (%MAF)**	Water-burst (%MAF)**	Unpurified (%MAF)***	Purified DNA (%MAF)**
1	74	F	Adenocarcinoma	Mod	*KRAS* G12V (11.6%)	45.1	17.4	No call	43.4	24.4	No call	39.4
2	50	M	Adenocarcinoma	Low	*KRAS* G12D (26.9%), *TP53* R273H (37.3%)	G12/G13 negative	31.1	No call	32.6	G12/G13 negative	No call	34.9
3	76	F	Adenocarcinoma	Low	*KRAS* G12R (27.4%), *TP53* R273C (33.8%)	44	30	No call	33.8	23.1	No call	14.7
4	59	F	Adenocarcinoma	Mod	*KRAS* G12D (24.8%), *TP53* I195T (32.4%)	20.5	40.9	No call	33.5	24.0	No call	28.6
5	74	M	Adenocarcinoma	Low	*KRAS* G12D (13.4%), *TP53* R273H (20.9%)	11.9	23.9	No call	29.5	21.3	No call	13.3
6	87	F	Adenocarcinoma	Low	*KRAS* G12C (43.8%), *CDKN2A* L63Q (24.1%), *TP53* H193L (22.9%)	18.9	36.9	No call	34.0	16.0	No call	48.0
7	76	M	Acinar cell carcinoma	High	No mutation call	0.27	G12/G13 negative	No call	G12/G13 negative	G12/G13 negative	No call	G12/G13 negative
8	65	F	Adenocarcinoma	Low	*KRAS* G12D (10%), *TP53* R213X (6%)	19.3	16.0	No call	27.9	G12/G13 negative	No call	19.0
9	43	M	Adenocarcinoma	Low	*KRAS* Q61R (7.8%)	G12/G13 negative (Q61 mutation; 10.4%)	G12/G13 negative (Q61 mutation; 18%)	No call	G12/G13 negative (Q61 mutation; 8%)	G12/G13 negative (Q61 mutation; 21%)	No call	G12/G13 negative (Q61 mutation; 6%)
10	69	M	Adenocarcinoma	Mod	*KRAS* G12V (8.2%), *TP53* R175H (12.6%)	33.8	42.0	No call	35.5	36.0	No call	37.0

*MAF* mutant allele frequency.

*Tumor cellularity; High, > 30%; Moderate (Mod), 10–30%; Low, < 10%.

**G12/G13 negative; *KRAS* G12/G13 mutants had a subthreshold prevalence (below 0.2%) relative to the wild-type.

***No call; Neither *KRAS* mutant nor wild-type allele was detected by digital PCR.

To confirm the fact that the *KRAS* mutations identified using this method originated from the tumor, pathological specimens from FFPE blocks were genotyped via targeted amplicon sequencing. In all 9 samples in which mutant *KRAS* was detected by the “water-burst” method, the *KRAS* mutation was pathologically proven to be present in the PDA tissue. In one patient with a *KRAS* G12D tumor, we failed to detect mutations during the FNA-needle flush process, while we could identify the mutation using DNA that was purified and concentrated from the supernatant and water-bursting of the residual tissue in FNA-needles (Table 2; patient 8). Another patient with an absent mutation calling in *KRAS,* as observed by the “water-burst” dPCR assay, was pathologically diagnosed as having a pancreatic acinar cell carcinoma with no *KRAS* mutations (patient 7). Taken together, the mutation detection method, and rapid and easy sample preparation rendered it highly feasible for us to identify *KRAS* mutations in small amounts of tissues.

## Discussion

The genetic profiling of solid tumors enables us to understand the molecular signatures of tumor development and progression more effectively; it also provides clinically relevant information for an early diagnosis, and pharmacological vulnerability and resistance towards various types of cancer^8^. The safer acquisition of cancer cells or tissue sampling sometimes makes it difficult for pathologists to secure a proper diagnosis. Recently, genetic tests utilizing a biopsy specimen have been more commonly used for patients with lung and colorectal cancer, for the selection of chemotherapeutic reagents; this generally requires a certain amount of tissues with a high tumor-cell content^14,15^. However, there are cases where a low tumor cellularity, as well as a tiny amount of tumor specimens per se hampered molecular analysis^16,17^. Besides, although DNA extraction/purification has been routinely performed for genetic testing, the process is time- and cost-consuming and sometimes significantly dilutes the target molecule. Here, we used specimens from patients with pancreatic cancer, which is characterized by a very low tumor cell content and abundant desmoplasia; these are challenging biospecimens not only for conventional immunohistochemistry analysis, but also for molecular analysis.

In this study, we evaluated a DNA preparation method without the purification step, for the genetic testing of the FNA specimen obtained from the pancreas, using the dPCR platform. We tried two different methods; the “cell-in-droplet” method involved the direct enclosure of the target cells into the droplet, followed by dPCR, while the “water-burst” approach attempted to capture tumor-derived DNA, following the osmotic burst of cancer cells, by their exposure to pure water just before their compartmentalization during the dPCR. We found that the latter approach was superior to the “cell-in-droplet” method. In the “water-burst” method, we could detect even a small number of cells with a homozygous *KRAS* mutation at codon 12, in as low as 20 cells (= 40 copies) in 4,000 normal cells with wild-type *KRAS*, showing that it was feasible to detect dPCR-based direct driver mutations in crude tumor tissues. We found this method to be clinically relevant, as it demonstrated that the *KRAS* mutation was detected in needle aspirates, with the tumor lesion cells being detected in 11 of 12 needle aspirates obtained from surgically resected pancreatic tissues, and in 9 of 10 residual tumor cells obtained from FNA needles, after sending the core specimens to the pathology lab. These results indicated that the combination of the “water-burst” approach and dPCR technology has the potential for detecting mutations in a super-sensitive manner, in a specimen with low-tumor cell content, such as a pancreatic tumor.

FNA is the gold standard for pathological diagnosis in patients with different types of cancer, including PDA, owing to its high diagnostic accuracy^18^. Because of tumor heterogeneity, multiple punctures might be required to avoid failure during pathological assessment. Occasionally, the report was based on the assessment of a limited number of cancer cells, and the use of insufficient amounts of tumor tissues for sampling can result in false-negative results. On the other hand, the dilemma associated with FNA involves the potential risk of bleeding and needle tract seeding at the puncture site^3, 19^. The detection of genetic mutations might compensate for limitations in pathological assessment, and the utility of such a strategy has been demonstrated^13,20^.

Next-generation sequencing (NGS)-based gene panel testing has been an invaluable tool in cancer diagnostics^7,21^. This modality offers a great deal of information related to genetic variation from a single sample, and over time, it has become much easier to operate. Nevertheless, a certain amount of high-quality DNA from an abundant of tumor tissue requiring multiple FNA punctures is required. Besides, the handling duration for the sample preparation, library quantification, and sequencing was long^22^. The limit of detection of mutations is > 1%, unless additional library preparation processes, such as molecular barcoding are employed, which would make the assay more expensive and time-consuming. Besides, careful bioinformatics assessments, such as those for error elimination and reporting are required to translate the data to the clinic^23^. On the other hand, the dPCR assay requires only a small amount of sample (1–5 ng of DNA), and the frequency of detected mutations is as low as 0.05%^24^. The running cost for dPCR is affordable, and it serves as an excellent filter for identifying high-risk patients.

The most distinctive feature of this study was that we could save on the effort, cost, and time required for DNA purification by simply suspending the stored material in water and breaking the cells. Molecular tests involving dPCR have not been used widely in the clinic^25^. This new method would potentially play an active, significant role in routine examinations. Because of the ease of sample preparation, operations with a high mobility during testing caused the confinement of regions of genes in a small number of samples, such as that observed during the compensatory assessment using the dPCR-method for the cytopathology test. The only parameter to ensure sample quality in the water-burst method is currently the copy number of *KRAS* amplified. A strong correlation was observed between the copy number and the amount of template DNA when the purification step was included in the same sample sets. Additional parameters such as DNA fragment size may help to precisely determine the quality of the crude samples.

We used a commercially validated screening probe set for detecting multiple *KRAS* codon 12/13 mutations using a small amount of tissue sample. There are several limitations associated with using this probe set. The first is that in this study, false positives (0.27% in FNA residual tissues) were observed. The threshold of the mutation frequency determined by the assay manufacturer was 0.2%. In the crude DNA used in this method, impurities existed or DNA was fragmented, because the degrading enzymes secreted from cells might have resulted in non-specific signals for mutations. In the future, it would become necessary to determine the cut-off value unique to our method, by using a larger number of tumor specimens in clinical settings.

The second issue was that screening probe set we utilized could detect multiple *KRAS* mutations at codons 12, 13, or 61; this does not provide accurate information associated with specific variations in mutations. Pancreatic neoplasia has often evolved with various distributed clonal backgrounds^8,26^; therefore, it is essential to determine each mutation pattern, to accurately identify primary lesions or the existence of coexisting malignant or benign lesions. Besides, a mutation in *KRAS* alone is not sufficient to provide genetic evidence of pancreatic cancer. Therefore, improvement of the current protocol targeting related mutations in other driver genes and tumor suppressors, such as *TP53* and *SMAD4*, is warranted. To solve this problem, we are currently developing a novel multiplex analysis method that identifies major *KRAS* and other gene mutations using 2D-spatial information regarding fluorescence intensity in dPCR. The dPCR system we used can distinguish two fluorescent colors^27^; however, a novel dPCR platform would be capable of simultaneously detecting multi-color dyes. Such a new tool might further enhance the utility of the assay during multiplex analysis, potentially allowing the detection of driver mutations across multiple genomic regions^28^.

The number of patients included in this study was minimal. Still, to further validate the feasibility for clinical use, it would be necessary to conduct clinical studies with a larger number of patient samples, and test various types of pancreatic tissues using FNA, ranging from benign to malignant tumor tissues. In addition to pancreatic cancer, validation studies are necessary for detecting driver mutations unique to other types of carcinomas, such as the *BRAF* V600E mutation observed during thyroid cancer^29^, as it would enhance the possibility of developing widespread clinical applications. Specifically, this approach would be clinically relevant for the minimally invasive pathological diagnosis of the tumor with a limited amount of tissue used for sampling. As observed during optional assessments using immunohistochemistry, the direct amplification and detection of key driver mutations would compensate for conventional pathological diagnosis using tissue biopsy and cytopathological analysis. Additional dPCR-based assessment of microsatellite instability may provide more detailed information regarding not only cancer diagnosis but also therapeutic implications.

In conclusion, we developed a digital PCR-based, super-sensitive assay for detecting mutations, which might resolve an issue related to the insufficiency of materials during cytopathological analysis. Our results indicated that a high rate of detection of *KRAS* mutations was associated with small amounts of FNA residual samples. Furthermore, using our “water-burst” method, we showed that even the DNA purification step was not necessary for detecting gene mutations in digital PCR. The straightforward and rapid protocol enables us to perform minimally invasive molecular analysis in cancer clinics.

## Methods

### Cell lines

Human pancreatic cancer cells (MIA PaCa-2; RCB2094) and non-cancer skin fibroblasts (NB1RGB; RCB0222) were obtained from the RIKEN cell bank (JAPAN), and grown using DMEM (MIA PaCa-2) and α-MEM (NB1RGB) media (FUJIFILM Wako chemicals, Japan) supplemented with 10% fetal bovine serum (GE Healthcare, Chicago, Illinois, USA) and 100 U/mL penicillin–streptomycin (FUJIFILM Wako chemicals). Cell lines were grown at 37 °C with 5% CO_2_ and passaged at 70–80% confluence. The number of cells was counted using the Countess automated cell counter (Thermo Fisher Scientific, Waltham, MA, USA).

### Patients

To examine the method for detecting mutations using resected tissues, twelve patients with the resectable pancreatic disease admitted in the Sapporo Higashi Tokushukai Hospital between 2017 and 2018 were included. Ten patients from whom FNA residual samples were obtained were recruited from Asahikawa Medical University in 2019. The study protocol for patient tissue collection and scientific analysis was approved by the Tokushukai Group Ethical Committee on Human Research (#TGE00357-012) and Asahikawa Medical University Research Ethics Committee (#17002). The study was conducted in accordance with the Declaration of Helsinki. Written informed consent was obtained from all patients before enrolment.

### Fresh tissue collection and preparation from surgically resected specimens

Small tissue specimens were obtained within 30 min after the surgical resection of the PDA in the operation room. Multiple tumor areas (typically, 2–3 areas) were punctured and aspirated using 22-gauge cathelin needles (TERUMO, Tokyo, Japan) connected to 10 mL syringes. The aspirated specimens were suspended in 3 mL of phosphate-buffered saline (PBS) containing 450 μL of stock solution from the PAXgene Blood ccfDNA Tube (BD Life Sciences; Franklin Lakes, NJ, USA) and stored for up to a week at 4 °C. The suspensions were centrifuged at 1,000×*g* for 10 min at room temperature, and the pellet was resuspended with 12 μL nuclease-free water, using a 200 μL pipette tip with a cut tip; this was immediately utilized as a PCR template.

### Collection of FNA-residual specimens

After performing FNA-biopsy sampling for cytological diagnosis, FNA residual tissues were obtained using a 22-gauge Franseen biopsy needle (Acquire; Boston Scientific, Marlborough, MA, USA). Residual tissues that remained in the needle were collected in a 5.0 mL microtube by performing aspiration several times (typically, 2–3 times), followed by the emission of 3 mL of physiological saline solution, which was combined with 450 μL of stock solution from a PAXgene Blood ccfDNA Tube^30^ by performing inversion and mixing several times and storing the contents for up to a week at 4 °C. Also, we collected needle-wash fluid fractions, to collect the washout tissues. Each suspension was centrifuged at 1,000×*g* for 10 min at 4 °C. The residual pellet fraction was partly scratched and resuspended with 12 μL nuclease-free water using a 200 μL pipette tip with a cut tip, and the entire pellet of the needle-wash fraction was resuspended in 12 μL nuclease-free water. The fraction resuspended in water was directly utilized as a dPCR template. The supernatant fraction obtained after centrifugation was directly input during dPCR. Purified DNA was prepared and used as a control in conventional mutation analysis methods. DNA in the supernatant fraction was purified using a QIAmp MinElute ccfDNA Mini Kit (Qiagen, Hilden, Germany), and the DNA in the pellet fraction was purified with a DNeasy Blood and Tissue Kit (Qiagen).

### Mutation detection assay using dPCR

Twenty microliters of the resuspension was mixed with 10 µL of ddPCR Supermix for Probes (no dUTP; Bio-Rad, Hercules, CA, USA), and 1 µL of ddPCR KRAS Screening Multiplex Kit that targeted *KRAS* exon 2 (#1863506; Bio-Rad) and template DNA solution, and then the mixture was vortexed three times at 2,500 rpm for 1 s. The PCR mixture was mixed with 70 µL Droplet Generation Oil (Bio-Rad) and compartmentalized using a QX200 droplet generator (Bio-Rad). The kit enables us to screen seven *KRAS* mutations (G12A/C/D/R/S/V and G13D) with a frequency > 0.2%, but specific variants cannot be determined. In the case of a tumor harboring *KRAS* Q61 mutation, as determined via NGS, an additional assay was performed using the ddPCR *KRAS* Q61 Screening Kit (Q61K/L/R/H, Bio-Rad), to evaluate the mutation status, with a cut-off > 0.5%. Specific variants also cannot be determined.

These mutation detection assays were performed using the following protocol: 10 min at 95 °C, followed by 40 cycles of 30 s at 94 °C, and 60 s at 55 °C, followed by a process for10 minutes at 98 °C (Ramp Rate; 2 °C/sec, at each step). The threshold for the absolute copy number input during the reaction and the ratio of the mutated fragments was calculated using QuantaSoft (ver 1.7; Bio-Rad), based on the Poisson distribution. Samples were scored as positive for mutant *KRAS* when at least five mutant droplets/reaction were detected using dPCR.

### Tumor specimens and mutation analysis

To validate the mutation signature of the tumor, formalin-fixed paraffin-embedded (FFPE) tissue specimens and unstained sections with a thickness of 10 μm or 4 μm (resected tissue or FNA biopsy specimen, respectively) were prepared. Genomic DNA was isolated using the GeneRead DNA FFPE Kit (Qiagen), and finally eluted with 30 μL of elution buffer, as described previously^31^. The purified DNA was quantified using the Qubit dsDNA HS Assay Kit on a Qubit4 fluorometer (Thermo Fisher Scientific).

Somatic mutations in the primary tumor of FFPE tissue specimens were also profiled using targeted amplicon sequencing techniques on the Ion AmpliSeq Custom Next-Generation Sequencing DNA panels, which were designed using the Ion AmpliSeq Designer Website (https://www.ampliseq.com), for targeting 8 PDA-related genes, namely *KRAS, TP53, SMAD4, CDKN2A, GNAS, PIK3CA, BRAF,* and *STK11* (Supplementary Table). Details regarding the sequencing analysis are described in the Supplementary Information.

## Supplementary information

Supplementary file1.

## Abbreviations

dPCR: Digital PCR
FNA: Fine-needle aspiration
PDA: Pancreatic ductal adenocarcinoma
