# Supplementary material for: Time-saving method for directly amplifying and capturing a minimal amount of pancreatic tumor-derived mutations from fine-needle aspirates using digital PCR
Source: Sci Rep. 2020 Jul 23;10:12332. doi: 10.1038/s41598-020-69221-6 (PMC7378187; doi:10.1038/s41598-020-69221-6)
Supplement: Supplementary file 1 — Supplementary file1. [file 41598_2020_69221_MOESM1_ESM.pdf]

Supplementary Information

Time-saving method for directly amplifying and capturing a minimal amount of pancreatic tumor-derived mutations from fine-needle aspirates using digital PCR

Yusuke Ono, Akihiro Hayashi, Chiho Maeda, Mayumi Suzuki, Reona Wada, Hiroki Sato, Hidemasa Kawabata, Tetsuhiro Okada, Takuma Goto, Hidenori Karasaki, Yusuke Mizukami, and Toshikatsu Okumura

This PDF file includes:

SI Materials and Methods

Fig. S1 and S2

Table S1 and S2

SI Materials and Methods

Targeted amplicon sequencing

A pancreatic ductal carcinomas (PDA)-associated gene panel (Ion AmpliSeq Custom DNA Panel) was designed using the Ion AmpliSeq Designer 3.6, to analyze the coding DNA sequences +25 bp away from the intronic flanking regions for 8 genes, namely *KRAS, TP53, SMAD4, CDKN2A, GNAS, PIK3CA, BRAF,* and *STK11*. The customized panel consisted of 42 amplicons in a single primer pool with a total of 4.7 kb DNA that covered 100% of the regions of interest, as shown in the BED file obtained from the Ion Ampliseq Cancer Hotspot Panel v2 (Thermo Fischer Scientific) (Supplementary Table). DNA (10–60 ng) was amplified using this panel, and a sequencing library was prepared. Sequencing and data analyses were performed using the Ion S5 GeneStudio system (Thermo Fisher Scientific, Waltham, MA, USA) as described previously [1]. Sequenced reads were demultiplexed, quality-filtered, and aligned to the human reference genome (GRCh37), using the Torrent Suite software package (ver. 5.0.4; Thermo Fisher Scientific). The mapping module (Torrent Mapping Alignment Program) is a sequence alignment software program optimized specifically for Ion Torrent data and includes several mapping algorithms with specific applications. Mapping was performed using default parameter values. Variants were identified using the Variant Caller plugin (ver. 5.0.4.0; Thermo Fisher Scientific) and included in the Torrent Suite Package, which was optimized to exploit the underlying flow signals. Variant calling analysis was performed using the somatic variant calling mode, which was optimized to detect low-frequency variants. The set parameters were as follows: minimum allele frequency, 0.02; minimum coverage, 100. To identify somatic mutations, the independent genotyping results for tumor and normal samples were excluded, and

variants in the normal samples were excluded from the molecular profile. Putative false-negative variants were excluded by analyzing the Phred-scale quality score, which was calculated using this plugin and manually confirming the alignment with IGV software (version 2.3.59; http://software.broadinstitute.org/software/igv/).

For variants containing novel exonic, nonsynonymous, and frameshift variants as well as intronic splice variants, the COSMIC (http://cancer.sanger.ac.uk/cosmic) and ClinVar databases (https://www.ncbi.nlm.nih.gov/clinvar/) were used for classification as either pathogenic or a variants with an unknown significance [2].

SI References

1 Nagai, K. et al. Metachronous intraductal papillary mucinous neoplasms disseminate via the pancreatic duct following resection. *Mod Pathol* (2019).

2 Omori, Y. et al. Pathways of progression from intraductal papillary mucinous neoplasm to pancreatic ductal adenocarcinoma based on molecular features. *Gastroenterology* **156,** 647-661 e642 (2019).

Supplementary Figure 1: dPCR results for the resected pancreata

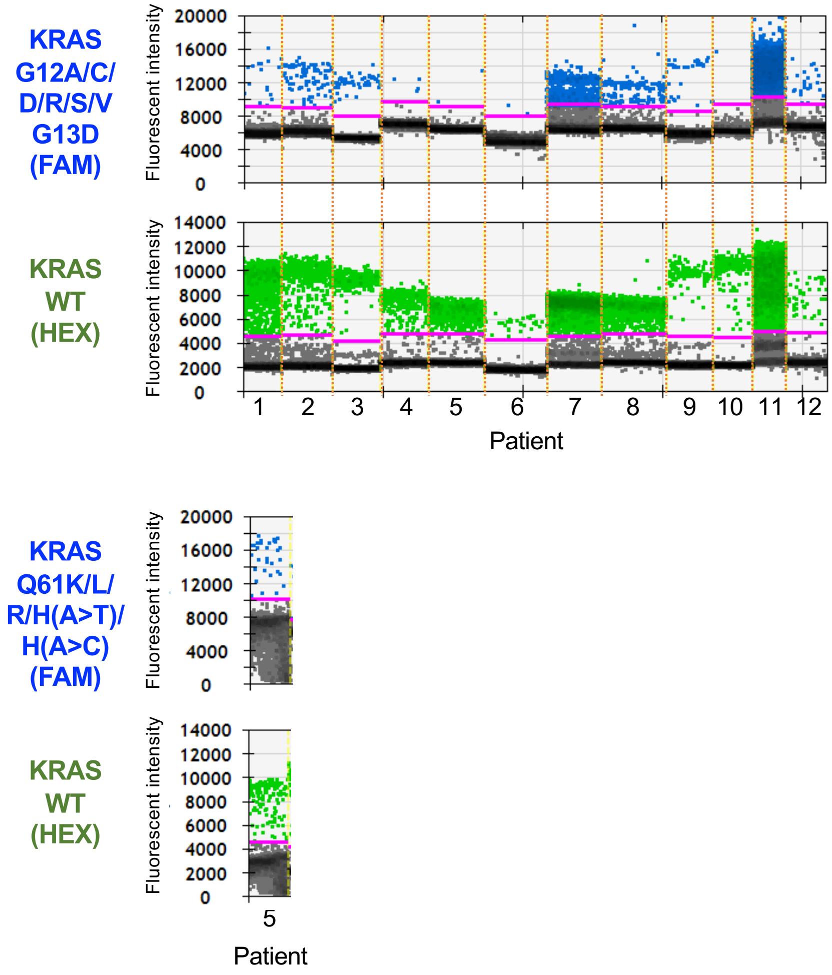

Twenty microliters of the resuspension was mixed with 10 µL of ddPCR Supermix for Probes (Bio-Rad) and 1 µL of ddPCR *KRAS* Screening Multiplex Kit (targets for G12A/C/D/R/S/V and G13D), and vortexed. After the reaction mixture was encapsulated in droplets using the QX200 droplet generator (Bio-Rad), PCR was performed; the endpoint fluorescence intensity of each droplet was counted and the copy number was calculated using QuantaSoft (ver 1.7; Bio-Rad), which was based on the Poisson distribution. The threshold line (solid pink line) was manually set to extend 2,000 or 1,000 amplitude (FAM mutant probe or HEX wild-type probe) above the maximum value for the background intensity. Table 1 shows the numerical data for dPCR and clinical profiles for each patient.

Supplementary Figure 2: Correlation between amplifiable DNA indexed by the copy number of *KRAS* and the amount of template DNA

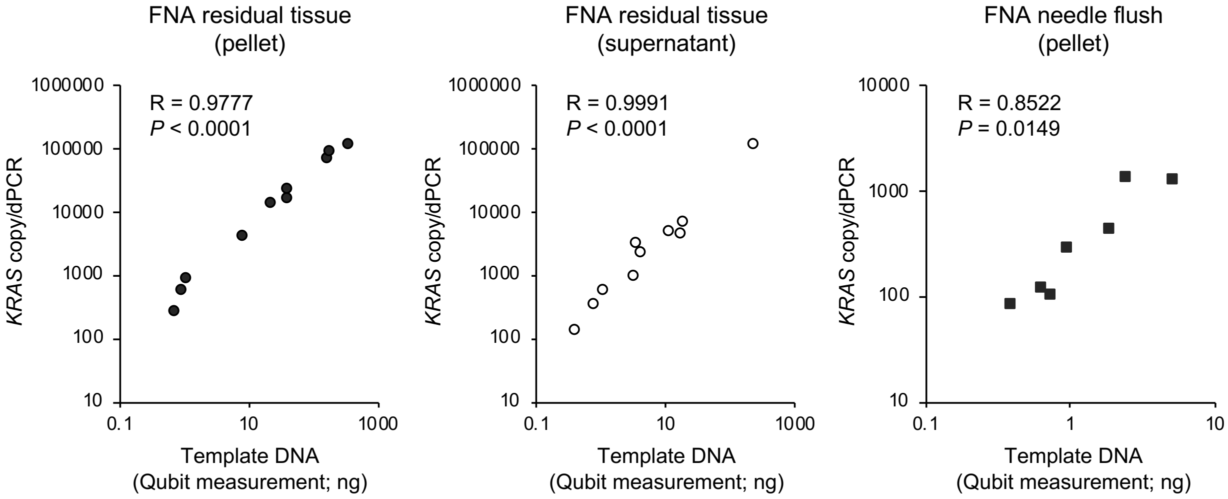

Correlation between amplifiable DNA indexed by the copy number of *KRAS* from FNA samples and the amount of template DNA quantified using a Qubit fluorometer. Pearson's correlation coefficients and *P*-values are shown for FNA residual tissue and needle flush (see Supplementary Table 2 for more details).

Supplementary Table 1: Covered region of NGS panel used in this study

Amplicon No.	Chromosome	Start	End	Gene
1	chr3	178916775	178916881	PIK3CA
2	chr3	178916931	178917035	PIK3CA
3	chr3	178921464	178921570	PIK3CA
4	chr3	178927405	178927525	PIK3CA
5	chr3	178927901	178927986	PIK3CA
6	chr3	178928069	178928160	PIK3CA
7	chr3	178936023	178936105	PIK3CA
8	chr3	178938787	178938918	PIK3CA
9	chr3	178947818	178947896	PIK3CA
10	chr3	178951996	178952097	PIK3CA
11	chr3	178952140	178952237	PIK3CA
12	chr7	140453102	140453221	BRAF
13	chr7	140481391	140481515	BRAF
14	chr9	21970940	21971066	CDKN2A
15	chr9	21971090	21971219	CDKN2A
16	chr12	25378549	25378658	KRAS
17	chr12	25380260	25380364	KRAS
18	chr12	25398186	25398304	KRAS
19	chr17	7573923	7574035	TP53
20	chr17	7577015	7577151	TP53
21	chr17	7577508	7577612	TP53
22	chr17	7578180	7578298	TP53
23	chr17	7578352	7578483	TP53
24	chr17	7578516	7578601	TP53
25	chr17	7579350	7579485	TP53
26	chr17	7579853	7579960	TP53
27	chr18	48575099	48575213	SMAD4
28	chr18	48575556	48575677	SMAD4
29	chr18	48581190	48581302	SMAD4
30	chr18	48584551	48584678	SMAD4
31	chr18	48586251	48586361	SMAD4
32	chr18	48591814	48591931	SMAD4
33	chr18	48593399	48593519	SMAD4
34	chr18	48603028	48603119	SMAD4
35	chr18	48604658	48604774	SMAD4
36	chr19	1206977	1207104	STK11
37	chr19	1220310	1220450	STK11
38	chr19	1220480	1220603	STK11
39	chr19	1221236	1221332	STK11
40	chr19	1223014	1223144	STK11
41	chr20	57484396	57484504	GNAS
42	chr20	57484562	57484672	GNAS

Sequencing DNA panels, which were custom designed to target the 8 PDA-related genes (*KRAS, TP53, SMAD4, CDKN2A, GNAS, PIK3CA, BRAF,* and *STK11*), consisted of 42 amplicons, with a total of 4.7 kb of DNA.

Supplementary Table 2: Copy number of *KRAS* amplified using dPCR assay and concentration of purified DNA from FNA residual tissues end needle flush

Patient	FNA residual tissue										FNA needle flush				
	Pellet					Supernatant					Pellet		Supernatant		
	Water-burst		Purified DNA			Water-burst		Purified DNA			Water-burst		Purified DNA		
	wild-type *KRAS* (copies/reaction)	mutant *KRAS* (copies/reaction)	Amount of template DNA (ng)	wild-type *KRAS* (copies/reaction)	mutant *KRAS* (copies/reaction)	wild-type *KRAS* (copies/reaction)	mutant *KRAS* (copies/reaction)	Amount of template DNA (ng)	wild-type *KRAS* (copies/reaction)	mutant *KRAS* (copies/reaction)	wild-type *KRAS* (copies/reaction)	mutant *KRAS* (copies/reaction)	Amount of template DNA (ng)	wild-type *KRAS* (copies/reaction)	mutant*KRAS* (copies/reaction)
1	292	94	43.6	12,900	2,720	no call	no call	12.06	2,740	2,060	78	64	5.382	762	466
2	no call	no call	182	45,000	20,200	no call	no call	234	77,800	28,700	no call	no call	2.502	850	460
3	164	130	376.8	76,200	32,600	no call	no call	19.35	4,420	2,260	11.4	38	0.765	86	15
4	260	66	23.4	7,820	5,420	no call	no call	1.143	350	176	76	24	N/A	280	112
5	112	15.2	8.631	3,080	966	no call	no call	3.636	2,120	888	42	11.4	N/A	130	20
6	770	159.2	1.161	538	316	no call	no call	0.774	220	110	64	12.2	0.657	60	54
7	3366	10.92	43.6	21,560	no call	no call	no call	3.276	884	no call	7	no call	N/A	24	no call
8	78.6	12.4	0.756	208	40	no call	no call	4.086	1,480	572	12	no call	N/A	222	50
9	230<br>(302)	no call<br>(36.8)	0.952	548.6<br>(366.6)	no call<br>(78)	no call<br>(no call)	no call<br>(no call)	0.424	126<br>(130)	no call<br>(11)	440<br>(152)	2.8<br>(42)	0.408	80<br>(92)	no call<br>(5.8)
10	8900	4460	188	47,040	34,650	no call	no call	16.92	2,680	1,434	270	158	1.9	270	158

Note: no call; Neither *KRAS* mutant nor wild-type allele was detected by digital PCR. Parentheses indicate results of *KRAS* Q61 mutation assay (patient 9)
Abbreviations; N/A, data not available
